# Population measles seroprevalence: Heterogeneity by birth-year cohort

**DOI:** 10.1016/j.jve.2023.100352

**Published:** 2023-10-09

**Authors:** Eduardo Santacruz-Sanmartin, Doracelly Hincapié-Palacio, Jesús Ochoa, Seti Buitrago, Marta Ospina

**Affiliations:** a Epidemiology Research Group in “Héctor Abad Gómez” National Faculty of Public Health at University of Antioquia, St 62 # 52-59, Medellín, Colombia; b Departmental Laboratory of Public Health- the Sectional Secretariat of Health and Social Protection of Antioquia, St 72 A # 78 B 141, Third Floor, Medellín, Colombia

**Keywords:** Measles, Seroprevalence, Finite mixture model, Colombia

## Abstract

**Objective:**

This work sought to estimate population measles seroprevalence and heterogeneity in the antibody concentration distribution that could be explained by the birth-year cohort according to the opportunity of viral and vaccine exposure, applied to data from Medellín, Colombia.

**Methods:**

Prevalence of IgG antibodies was analyzed for measles based on a population study with a random sample of 2098 individuals from 6 to 64 years of age. Finite mixture models were used to estimate global seroprevalence and that of three birth-year cohorts (I: born up to 1982; II: 1983–1994; III: born since 1995). Multiple linear regression permitted adjusting the concentration of antibodies by cohort, zone, and sex.

**Results:**

Globally, seronegativity was 6.5% (95% CI 4.9– 8.6), seropositivity of 78.4% (95% CI 75.1–81.4), and equivocal of 15.1% (95% CI 12.5–18.1). Two components were found with skewed normal distribution, which reclassified those equivocal as seropositive. Differences were observed by cohort in the geometric mean of antibodies [Cohort I: 1704.6; II: 562.2; III: 802.1 milli-international units per milliliter (mIU/mL] and seronegativity (Cohort I: 4%; II:13.3%; III: 8.9%). Antibody concentration increased by 1.26 mIU/mL in residents in the rural area, while diminishing in individuals from cohort II (by 3.02 mIU/mL) and cohort III (by 2.14 mIU/mL).

**Conclusion:**

The younger cohorts (II and III) had a lower antibody concentration (higher seronegativity), indicating the need to monitor periodically seroprevalence and an eventual reestablishment of the transmission in these groups with higher risk of infection.

## 1 Introduction

Prior to the introduction of the vaccine against measles in the 1960s, globally, there were over 2-million deaths per year attributed to measles.^1^ Currently, measles continues being a public health problem, causing over 100,000 deaths annually.^2^

Since 2008, none of the regions of the World Health Organization (WHO) has maintained vaccination coverage for measles-containing-vaccine first-dose (MCV1) above 95 % as required for herd immunity.^3^^,^^4^ This situation worsened after the Covid-19 pandemic,^4^, ^5^, ^6^ where the highest coverage in 2022 was reported in regions, such as Europe (93%), South East Asia (92 %), and Western Pacific (92%).^4^

Elimination of measles may be affected by the resurgence of cases registered in the last decade. In the world, reported cases diminished between 2000 and 2016 (853,479 cases in 2000 to 132,490 cases in 2016), but in 2019 it increased by 556% (869,770 cases).^2^ Outbreaks were reported in Africa, Europe, and the Americas.^2^ In the latter, the increase in reported cases was more noticeable, around 22,551%, going from 92 cases in 2016 to 19,244 cases in 2019.^7^ A consequence of that was the re-establishment of the endemic transmission in Venezuela in 2018 and Brazil in 2019.^2^^,^^8^

In Colombia, as in other countries in the region, accumulation of susceptible individuals (such as unvaccinated children) is concerning, which can influence the recent resurgence of the disease. In the country, outbreaks were reported in 2002, 2018, and 2019, with 139, 208, and 244 confirmed cases, respectively.^9^

Estimating seroprevalence of measles during a post-vaccine period in areas with certified elimination is useful.^10^ The WHO has insisted on the need to have good-quality data at subnational level to detect the accumulation of susceptible individuals and, thus, to prevent outbreak occurrences and resurgence of the disease.^11^ Often, data on vaccine coverages does not represent population immunity due to the inaccuracy of individual vaccination data and of the population census, added to the lack of monitoring of the vaccine effectiveness and lack of alerts of individuals vaccinated with incomplete schemes.^12^

Susceptibility detection is possible through serosurveillance of the immune profile of the population or of some subgroups. Periodic determination of susceptibility through hospital serosurveillance can be a novel mean of documenting progress in the elimination of measles, as an alternative to probabilistic surveys of homes that require time, resources, and logistic capacity.^13^

Serosurveillance could be used to guide vaccination programs with the aim of reducing/avoiding the occurrence of epidemics and disease resurgence,^14^ given that, with periodic application of serological surveys, changes in population immunity can be monitored^15^ and, in turn, provide guidance for local and global vaccination plans.^14^

A challenge to estimate measles seroprevalence is the lack of a correlate of protection, especially in regions in the elimination process.^16^^,^^17^ Some authors have proposed the hypothesis about the immunity of measles as a continuum more than a binary state, in which case finite mixture models become an analysis alternative.^18^

Finite mixture models help to identify heterogeneity in the antibody concentration distribution, explained by different degrees of exposure to a certain antigen due to exposure by age, vaccination opportunity, among other covariables.^18^ It has been suggested that using the manufacturer's cut-off values may cause classification errors of the serological status, while the finite mixture models permit estimating the proportion or probability of being seropositive or seronegative by using the full distribution of the antibody concentration.^19^

A finite mixture model was used to identify heterogeneity in antibody concentration distribution and population measles seroprevalence in Medellín, Colombia, which could be explained through the birth-year cohort, according to the opportunity of viral exposure and to the vaccination scheme against measles.

## 2 Methods

### 2.1 Study population

Prevalence of IgG antibodies against measles was estimated in residual samples from a random population study conducted in Medellín, Colombia in 2009 by age, sex, and zone (urban-rural).^20^ The samples were kept in the serum bank of the serosurveillance program of the Departmental Public Health Laboratory of the Sectional Secretary of Health and Social Protection of Antioquia where they were also processed.

The study was approved by the ethics committee of the Héctor Abad Gómez National Faculty of Public Health at Universidad de Antioquia (minutes 17 of 2007 and 204–01 Feb. 2019), in northwest Columbia.

### 2.2 Laboratory tests

Anti-measles IgG antibodies were measured with Enzygnost® Measles Enzyme-Linked Immune-Sorbent kit G (99.6% sensitivity and 100% specificity),^21^ following the manufacturer's recommendations. Antibody concentration was obtained using the alpha method expressed in milli-international units per milliliter (mIU/mL). Participants were considered “seronegative” if the antibody concentration was <150 mIU/mL. Above the antibody threshold of 150 mIU/mL, individuals were classified as equivocal (150–350 mIU/mL) and positive (>350 mIU/mL), according to the cut-off points established by the manufacturer.^10^

### 2.3 Statistical analysis

The demographic characteristics of the participants were analyzed using measures of central tendency, dispersion and position, like arithmetic mean (± standard deviation, SD), median, minimum and maximum in case of continuous variables. Categorical variables and seroprevalence were shown with absolute and relative frequencies.

Prevalence estimation was conducted by calculating weighted proportions from the effect of multistage survey design, using the cut-off points established by the manufacturer.

The history of vaccination against measles was established by cohorts, according to vaccination strategies in the country. Study participants had access to vaccination against measles during childhood and during adulthood, as well as a potential viral exposure. The vaccination was different by birth-year cohort due to strategies implemented in each period. In 1973, when the vaccination against measles was officially introduced in Colombia, it was applied in a monovalent manner.^22^^,^^23^ Since 1984, the regular vaccination strategy was complemented with update campaigns.^23^ Since 1995, the first dose of the triple viral vaccine (against Measles, Mumps and Rubella - MMR) is administered at 12 months of age. In 1997, an MMR booster was administered at 10 years of age, which since 2004 changed to 5 years of age.^24^ Between 1995 and 1997, vaccination campaigns administered MMR to populations born since 1984.^20^^,^^24^^,^^25^ During the years 2005–2006, a national vaccination campaign with a double viral vaccine (measles and rubella-MR) was carried out, aimed at men and women from 14 to 39 years of age.^20^

Logarithmic transformation was performed for the concentration of measles IgG antibodies. Serological data was analyzed using the fixed cut-off values according to the manufacturer's instructions of the laboratory test and a finite mixture model^26^ was applied under the assumption that the tested samples were taken from individuals with different immune states, each of which can describe the results of antibodies through some distribution.^19^^,^^27^

Based on the manufacturer's cut-off values of the test, three groups of results were obtained: positive, negative, and equivocal. The finite mixture model sought to identify the number of components or subpopulations that could reflect heterogeneity in exposure and its corresponding average of antibodies and seroprevalence proportion, globally and by birth-year cohort. The number of components was selected along with the type of probability distribution (normal, skewed normal, Student's t, skewed Student's t). The best fit was determined according to the Bayesian Information Criterion (BIC). Seropositivity and seronegativity were recalculated based on probability distribution data with best fit, calculating the mean plus two standard deviations and the 99% quantile.^28^ Subsequently, a multiple linear regression was estimated to determine the factors potentially associated with the concentration of measles IgG antibodies.^29^ Statistical significance was considered for p < 0.05.

The descriptive analysis was carried out using Stata16 (Stata Corporation, College Station, Texas, USA) and SPSS® version 25 (IBM SPSS Statistics for Windows, Armonk, NY: IBM Corp). RStudio 4.1.1 was used to estimate the finite mixture model with the mixmsn package^30^^,^^31^ and the multivariate model.

## 3 Results

### 3.1 Characteristics of participants according to level of measles IgG antibodies

Samples from 2098 individuals from 6 to 64 years of age were analyzed to determine measles IgG levels. The participants had a mean age of 30.9 years (SD 16.4). According with the cut-off values from the manufacturer of the laboratory test, the weighted seronegativity proportion was 6.5%, without differences for age, sex, zone of residence. In the age group from 41 to 64 years, a significant difference was observed in the proportion of equivocal and seropositive compared to the other groups (Table 1).

**Table 1:** Characteristics of participants according to sex, age group, and zone of residence. Medellín, 2009.

Characteristic		Level of measles IgG antibodies									Total
		Seronegative			Equivocal			Seropositive			
		N	%pond (95%CI)	Deff	n	%pond (95%CI)	Deff	n	%pond (95%CI)	Deff	
**Sex**											
	Male	57	5.8 (3.6–9.4)	3.8	121	17.1 (13.3–21.8)	3.3	642	77.0 (71.9–81.5)	3.4	820
	Female	101	7.2 (5.2–9.9)	2.3	144	13.2 (10.1–17.0)	2.9	1033	79.6 (75.6–83.2)	2.5	1278
**Age group (years)**											
	41–64	16	1.6 (0.7–3.9)	1.8	20	**4.2 (2.0**–**8.5)**	3.2	616	**94.2 (90.0**–**96.7)**	2.7	652
	18–40	69	6.0 (3.8–9.4)	2.7	136	19.5 (15.1–24.8)	3.2	669	74.5 (69.0–79.3)	3.0	874
	6–17	73	10.8 (7.4–15.7)	3.3	109	18.3 (13.4–24.3)	3.7	390	70.9 (64.3–76.7)	3.5	572
**Zone**											
	Urban	59	6.2 (4.5–8.4)	3.3	124	15.1 (12.3–18.3)	3.6	807	78.7 (75.2–81.9)	3.4	990
	Rural	99	12.4 (9.6–16.0)	0.3	141	15.2 (12.4–18.5)	0.2	868	72.4 (67.9–76.4)	0.3	1108
**Total**		**158**	**6.5 (4.9**–**8.6)**	**3.0**	**265**	**15.1 (12.5**–**18.1)**	**3.4**	**1675**	**78.4 (75.1**–**81.4)**	**3.2**	**2098**

n: number of cases; %pond: weighted proportion in percentage; Deff: design effect.

### 3.2 Measles IgG antibodies

The distribution of measles IgG antibody titers in milli international units per milliliter (mIU/mL) did not distribute normally (p value of Kolmogorov Smirnov test <0.05, kurtosis value > 0) when analyzed globally and for the birth-year cohort I; for cohorts II and III, the assumption of normality was fulfilled, although in this last cohort at a limit value (p value of Kolmogorov-Smirnov test = 0.05). Given this situation, the geometric mean is taken as a summary measure, where higher titers were observed in older people (Cohort I), followed by the youngest cohort (Cohort III) and, lastly, the intermediate cohort. The value of the median of antibodies lower than the mean value indicates a distribution of titers skewed to the right (Table 2).

**Table 2:** Descriptive indicators of the measles IgG antibody titers by birth-year cohort according to vaccination scheme. Medellín, 2009.

Birth-year cohort according to vaccination scheme^a^	Mean^b^	Standard deviation^b^	Median^b^	Geometric mean^b^	Kurtosis	P value (K-Smirnov)^c^	n
Cohort I (1944–1982)	2838.5	2456.3	2184.4	1704.6	1.90	0.00	1125
Cohort II (1983–1994)	1133.3	1610.4	557.9	562.2	−0.10	0.20	601
Cohort III (1995–2003)	1555.1	1831.7	860.1	802.1	0.13	0.05	372
**Total**	**2122.5**	**2276.5**	**1196.7**	**1085.3**	**0.11**	**0.00**	**2098**

a Cohort I: born until 1982, start of massive immunization, natural exposure to the virus, or who received at least one dose of measles-containing vaccine (MCV); cohort II: born between 1983 and 1994: MCV in regular program plus “catch-up” campaigns (1984), Measles Mumps and Rubella-MMR (1995–1997) and MCV (2005); cohort III: born since 1995, 2-dose MMR.

b Milli-international units per milliliter (mIU/mL).

c Kolmogorov Smirnov normality test with Lilliefors correctionn: number of cases.

### 3.3 Level of measles IgG antibodies by birth-year cohort according to vaccination scheme

**Fig. 1:**
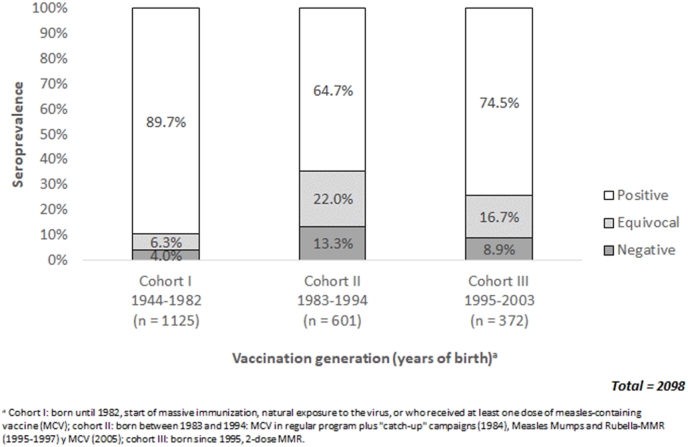
Level of Measles IgG antibodies by birth-year cohort according to vaccination scheme.

According with the cut-off values from the manufacturer of the laboratory test, the sample data showed that the highest number of seronegative cases belonged to people from cohort II, followed by cohort III and with lower number in cohort I (older people) (Fig. 1).

### 3.4 Finite mixture models

The analysis was performed with the logarithmic transformation in base 10 of the measles IgG antibodies (Log10). The number of components in the mixture models was allowed to vary from 1 to 4. For the entire sample, it was observed that it can be divided into two principal classes, with evidence of two serological subpopulations. According with the BIC, the best model for the total data was the skewed normal distribution, with a Log10 of the antibody mean of 3.2 (SD 0.7) and 3.5 (SD 0.2), equivalent to 1584.9 mIU/mL and 3162.3 mIU/mL in each group, respectively (Table 3).

**Table 3:** Finite mixture models for measles IgG antibodies. Medellín, 2009.

Type of distribution	Components	BIC	Group 1^a^		Group 2^a^		Group 3^a^	
			Mean	Variance	Mean	Variance	Mean	Variance
**Skewed normal**	1	3458.6						
	**2**	**3418.7**	**3.2**	**0.46**	**3.5**	**0.05**		
	3	3443.7						
	4	3465.0						
Normal	1	3622.4						
	2	3448.0						
	3	3424.7	3.6	0.04	2.9	0.14	2.5	0.32
	4	3433.7						
Skewed Student's t	1	3473.9						
	2	3425.6	3.2	0.34	3.6	0.04		
	3	3455.6						
	4	3471.1						
Student's t	1	3629.5						
	2	3442.5						
	3	3438.9	2.5	0.21	3.0	0.10	3.6	0.04
	4	3441.6						

a Values in Log base 10 for measles IgG antibody titers.

### 3.5 Distribution of antibodies from the finite mixture model

**Fig. 2:**
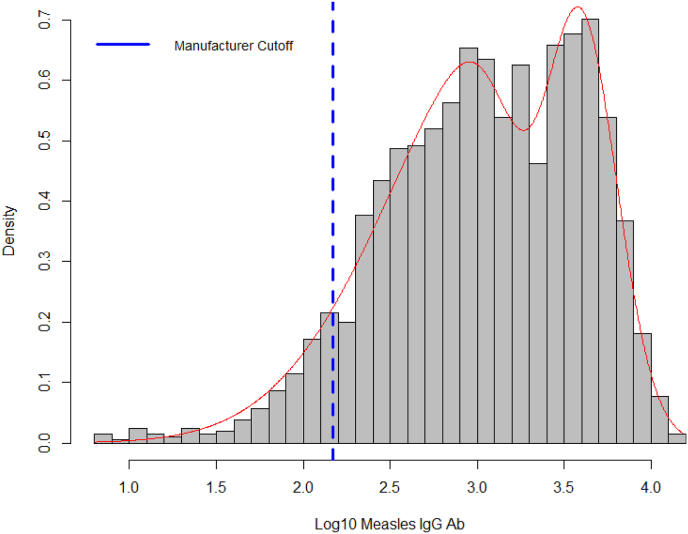
Distribution of IgG antibodies for measles. Medellín, 2009.

The behavior of the antibodies transformed showed distribution skewed to the right, with a better fit than the skewed normal distribution (Fig. 2).

Upon calculating the mean plus two standard deviations and the 99% quantile, values were obtained of 2.50 and 2.17 (Log10 IgG), respectively, showing similarity between the manufacturer's cut-off value and the results from the finite mixture model. Thus, equivocal titers were reclassified as seropositive.

The finite mixture analysis of cohort I identified a single component. According with the BIC, the best model for this cohort is the skewed normal distribution, with a Log10 of the antibody mean of 3.9 (SD 0.8), equivalent to 7943.3 mIU/mL (Table 4).

**Table 4:** Finite mixture models for measles IgG antibodies for cohort I. Medellín, 2009.

Type of distribution	Components	BIC	Group 1^a^		Group 2^a^		Group 3^a^		Group 4^a^	
			Mean	Variance	Mean	Variance	Mean	Variance	Mean	Variance
**Skewed normal**	**1**	**1510.2**	**3.9**	**0.68**						
	2	1536.0								
	3	1554.7								
	4	1564.4								
Normal	1	1763.5								
	2	1583.8								
	3	1536.2	3.6	0.04	3.0	0.15	2.4	0.5		
	4	1544.6								
Skewed Student's t	1	1512.9	3.9	0.54						
	2	1539.6								
	3	1562.2								
	4	1571.3								
Student's t	1	1720.4								
	2	1560.9								
	3	1554.2								
	4	1551.1	2.7	0.10	1.9	0.47	3.2	0.05	3.7	0.03

a Values in Log base 10 for measles IgG antibody titers.

The finite mixture of cohort II identified a single component with better fit for the normal distribution according with the BIC, with a Log10 of the antibody mean of 2.7 (SD 0.5), equivalent to 127.0 mIU/mL (Table 5).

**Table 5:** Finite mixture models for measles IgG antibodies for cohort II. Medellín, 2009.

Type of distribution	Components	BIC	Group 1^a^	
			Mean	Variance
Skewed normal	1	947.6	3.1	0.39
	2	970.4		
	3	985.2		
	4	1009.3		
**Normal**	**1**	**940.3**	**2.7**	**0.27**
	2	960.0		
	3	967.2		
	4	984.1		

a Values in Log base 10 for measles IgG antibody titers.

In the finite mixture analysis, a single component was identified in cohort III. According with the BIC, the best model for this cohort was the skewed normal distribution, with a Log10 of the antibody mean of 3.4 (SD 0.7), equivalent to 2511.9 mIU/mL (Table 6).

**Table 6:** Finite mixture models for measles IgG antibodies for cohort III. Medellín, 2009.

Type of distribution	Components	BIC	Group 1^a^	
			Mean	Variance
**Skewed normal**	**1**	**613.7**	**3.4**	**0.50**
	2	634.1		
	3	651.8		
	4	663.7		
Normal	1	615.1	2.9	0.30
	2	625.3		
	3	635.4		
	4	646.0		
skew Student's t	1	620.2	3.3	0.47
	2	641.1		
	3	654.5		
	4	677.8		
Student's t	1	620.8	2.9	0.29
	2	629.8		
	3	637.1		
	4	654.5		

a Values in Log base 10 for measles IgG antibody titers.

### 3.6 Fit of antibody titers by birth-year cohort according to vaccination scheme from finite mixture models

**Fig. 3:**
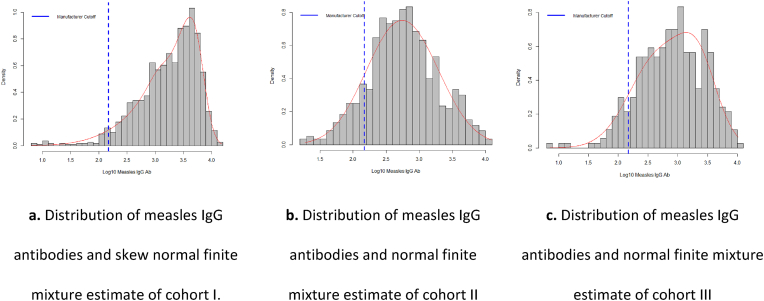
Distribution of measles IgG antibodies and estimate of finite mixture by birth-year cohort according to vaccination scheme. Medellín, 2009.

The Log10 behavior of antibodies in cohorts I and III showed distribution skewed to the right, with a better fit than the skewed normal distribution (Fig. 3a and c). For cohort II, greater symmetry was observed with a fit to the normal distribution (Fig. 3b).

### 3.7 Potential factors associated with the level of antibodies

A multiple linear regression model adjusted for covariates was estimated, obtaining a mean of measles IgG antibodies of 1479.1 mIU/mL (Log_10_ = 3.17), where birth-year cohort and rural zone were associated with antibody titers, but not sex. Belonging to a cohort different from 1 (until 1982) diminished titers: cohort 1983–1994 by 3.02 mIU/mL (Log_10_ = 0.48) and cohort 1995–2003 by 2.14 mIU/mL (Log_10_ = 0.33); being from rural area increased them by 1.26 mIU/mL (Log_10_ = 0.10). The adjusted coefficient of determination, R^2^ = 0.15, indicated that the birth-year cohort and rural zone of residence explained 15% of changes in antibody titers. According to the analysis of variance (ANOVA), this model was adequate (p < 0.05), given that at least one coefficient was different from zero. When this ANOVA was performed separately with each variable, the statistical significance of each was reaffirmed. No multicollinearity was observed given the absence of variables with tolerance <0.1 and VIF >10 (data not shown) (Table 7).

**Table 7:** Measles IgG antibody multiple linear regression model.

Coefficients:	Estimate	Std. Error	Confidence Interval of the coefficient		t value	P value
			2.5%	97.5%		
(Intercept)	3.19	0.02	3.15	3.22	167.78	0.00
Cohort II	−0.48	0.03	−0.53	−0.43	−18.12	0.00
Cohort III	−0.33	0.03	−0.39	−0.27	−10.52	0.00
Zone: Rural	0.10	0.02	0.05	0.14	4.32	0.00
Sex: Female	0.02	0.02	−0.01	0.02	0.72	0.47

Adjusted R-squared: 0.15.

F-statistic: 124.7 on 3 and 2094 DF, p-value: <2.2e-16.

Lilliefors (Kolmogorov-Smirnov) normality test data: residuals (model) D = 0.050845, p-value <1.003e-13.

a Cohort I: born until 1982, start of massive immunization, natural exposure to the virus, or who received at least one dose of measles-containing vaccine (MCV); cohort II: born between 1983 and 1994: MCV in regular program plus “catch-up” campaigns (1984), Measles Mumps and Rubella-MMR (1995–1997) and MCV (2005); cohort III: born since 1995, 2-dose MMR.

### 3.8 Residual analysis

This multiple linear regression model is explicative, residuals were not distributed normally (Kolmogorov-Smirnov normality test with Lilliefors correction p < 0.05) and no correlation was observed (Durbin Watson test p < 0.05) (Table 7).

## 4 Discussion

Our study estimated the population measles seroprevalence during a post-vaccine period, after 36 years from the start of mass vaccination, based on a random sample by age, sex, and zone of residence of the city of Medellín, Colombia.

Estimation of seroprevalence was based on the cut-off values established by the manufacturer, compared with the full distribution of the data using finite mixture models, especially useful for the need to characterize the measles seroprevalence profile.^17^

The seroprevalence weighted proportion of our study was lower than that reported in the study by Diaz-Ortega et al.*,* conducted in Mexico in 2012, who obtained a weighted prevalence of 99.37% (95% CI 99.07–99.58), using a plaque-reduction neutralization test.^32^ They also found higher susceptibility in the group from 30 to 39 years of age.^32^

In Colombia, few studies exist on population measles seroprevalence. In 2016, Gonzalez et al., conducted a population study of healthy children from 5 to 9 years of age, residing in nine municipalities of the department of Quindío, located, like Medellín, in the central zone of Colombia. The authors used a commercial ELISA test (Diagnostic automation INC® CA, USA) and the manufacturer's cut-off values. Based on a random sample of 170 individuals, they detected seroprevalence of 86.4 % (95% CI 80.18–91.05).^33^

This study found higher antibody levels in the cohort of people of greater ages (cohort I), which may be related with greater opportunity for natural infection, while the younger cohort (cohort III) had greater opportunity for exposure to vaccination than those in cohort I and even than the intermediate cohort (cohort II).^34^

The two components from the finite mixture model could represent the serological classification of seronegative and seropositive, although with a higher cut-off value than that proposed by the manufacturer of 150 mIU/mL. The skew to the right of the antibodies would indicate differential exposure by age, mainly due to exposure to the wild virus, above all in cohort I (older people).^35^^,^^36^

In our study, antibody concentration was associated with the birth-year cohort and zone of residence. Diaz-Ortega et al.*,* have observed as factors associated with susceptibility to measles at young ages, living in the country's capital, living in overcrowded conditions, and having an unknown vaccination stause or not having received a vaccine against measles between 1 and 5 years of age.^32^ In Haiti, in 2017, Minta et al., observed higher susceptibility in regions where vaccine coverages were lower, using a multiplex bead assay to determine antibodies against measles.^37^

Our study was limited by the lack of data about the history of disease and immunization of the participants. The analysis of the birth-year cohorts was an approach to understanding differences in the opportunity of exposure to the measles virus and different vaccination strategies.

The analysis of finite mixture models permitted reclassifying individuals considered equivocal, according to the manufacturer's cut-off values as seropositive without reprocessing the samples and in keeping with the literature.^16^ Estimating seropositivity using finite mixture models is indicated in this case by the lack of correlates of protection, which limits the use of the manufacturer's cut-off values, especially regarding heterogeneity in antibody concentration distribution, as observed in this case through birth-year cohort.^19^ Nonetheless, it would be required to determine if seronegativity correlates with susceptibility and, hence, if these individuals have protection against the disease by evaluating cell immunity.^16^

In conclusion, differences were observed in the distribution of IgG antibodies for measles by birth-year cohort, with a higher proportion of seropositivity in the older cohort(cohort I) who probably had higher natural exposure,^35^^,^^36^ while the younger cohorts (cohort II and III) had greater seronegativity.

It is recommended to periodically monitor population seroprevalence in the younger cohorts to corroborate the potential decrease in immunity over time and detect conglomerates of seronegative individuals that guide the identification of population groups with higher risk of infection and an eventual resurgence of the local transmission of measles.

## Funding

This work was funded by the Administrative Department of Science, Technology, and Innovation “COLCIENCIAS- MINCIENCIAS”- Colombia (Grant # 699-2018), Departmental Laboratory of Public Health of the Sectional Secretariat of Health and Social Protection of Antioquia, Secretariat of Health of Medellín, and Universidad de Antioquia, Medellín, Colombia.

## Declaration of competing interest

The authors declare that they have no known competing financial interests or personal relationships that could have appeared to influence the work reported in this paper.

## Data Availability

The authors do not have permission to share data.
